# The influence of a year-round tillage and residue management model on soil N fractions in a wheat-maize cropping system in central China

**DOI:** 10.1038/s41598-019-41409-5

**Published:** 2019-03-18

**Authors:** Xinxin Ye, Yin Ye, Rushan Chai, Junli Li, Chao Ma, Hongying Li, Qizhong Xiong, Hongjian Gao

**Affiliations:** 1 Anhui Province Key Laboratory of Farmland Ecological Conservation and Pollution Prevention, School of Resources and Environment, Anhui Agricultural University, Hefei, 230036 China; 2 Institute of Soil and Fertilizer, Anhui Academy of Agricultural Sciences, Hefei, 230031 China

## Abstract

Tillage practice and residue management play important roles in N pool in soils. This study determined the impacts of tillage practice and residue management on crop yield. It also investigated the distribution, fractionation, and stratification of N at soil at depths ranging from 0 to 60 cm under wheat–maize cropping systems. Three treatments were established in 2009: no-tillage with straw removal for winter wheat and summer maize (NT), no-tillage with straw mulching for winter wheat and summer maize (NTS), no-tillage with straw mulching for summer maize and plow tillage with straw incorporation for winter wheat (NPTS). After 8 years, soil total nitrogen (TN) content in NTS was greater than in NT, but only in 0–10 cm layer. NPTS treatment increased TN content over NT and NTS in 10–20 cm layer by 18.0% and 13.9%, and by 16.8% and 18.1% in 20–30 cm layer, respectively. Particulate organic N, microbial biomass N and water-extractable organic N levels were the greatest in 0–10 cm layer under NTS treatment; and in 10–30 cm layer, the corresponding values were the highest under NPTS treatment. NPTS treatment could immobilize the mineral N in 10–30 cm layer, and reduced leaching losses into deeper soil layers (40–60 cm). Furthermore, total yield increased by 14.7% and 8.5% in NPTS treatment compared to NT and NTS treatments, respectively. These results indicate that NPTS is an effective and sustainable management practice, which will improve soil fertility, sustainable crop production, and environmental quality in low-productivity soils in central China.

## Introduction

Nitrogen (N) is required if sustainable crop yields are to be achieved when intensive cropping systems are used on dry land^1^. In 2011, the total consumption of inorganic N fertilizers in China was about 36.9 million tones (Mt), which accounted for 35.1% of the global N consumption by agriculture^2^. The extensive application of chemical N fertilizers directly onto farmlands is generally accepted to have caused a number of environmental problems^3^. Better management of N in the soil–plant system is key to coordinating the relationships among crop yield, product profit, and environmental protection^4^. Therefore, it is important to further understand the N availability and fraction variations in soils under different management regimes.

The soil total nitrogen changes slowly when management practices change, but the labile fractions (the N fractions easily vary in soils) have been made to relate to plant available N. Many researchers have used particulate organic N (PON) as an index of the soil labile N pools because it is sensitive to soil disturbance and crop residue inputs^5^. Soil microbial biomass N (MBN) is very sensitive to alterations in soil management and is often used as a biological indicator of soil management changes^6^. The water-extractable organic N (WEON) is the primary energy source for soil microorganisms and is an indicator of nitrogen availability to soil microorganisms^7^. Therefore, the comparisons of soil N fractions in different management treatments provide an index of the status of N levels.

Crop residue management can influence soil N cycling. The addition of crop residues can improve soil quality and increase soil nutrients, especially N input^8^. Furthermore, crop residues are the primary energy resource for soil microorganisms and an important source of plant nutrients. Long-term retention rather than removal of crop residues from farmland with appropriate fertilization have been demonstrated to improve soil fertility and increase crop yields^9^. The application of straw residue has also been shown to immobilize mineral N and decrease its losses in soil^10^ due to chemical and biotic processes^11^, particularly the rapid increase in microbial immobilization of inorganic N fertilizer^12^. Therefore, crop residues are the energy and material sources for soil microorganisms and plants, and their retention may improve soil N cycling, and facilitate increases in crop yield and N uptake.

Tillage practice can also affect N availability and nitrogen storage in the soil due to its short-term and long-term influences on the physical, chemical, and biological properties of the soil^13^. Conventional tillage favors the decomposition of crop residues and soil organic matter (SOM) by enhancing aeration and promoting microbial activity in the soil, which increase C and N cycling^14^. In addition, tillage practices distribute organic carbon and nutrient sources more uniformly in the soil profile^15^. No-tillage systems produce less soil disturbance, which saves energy, improves soil quality, and maintains soil fertility^16^. Therefore, the selection of proper tillage practices, based on the soil environmental status, can improve resource use efficiency and crop yields.

Lime concretion black soil, which is derived from fluvial-lacustrine sedimentation, is one of the Calcic Vertisol soils according to the World Reference Base for Soil Resources (WRB 2006). This soil is a typical low-yielding soil in China due to its high clay content and poor soil structure^17^. It is widely distributed in central China and covers about 2.98 million ha, but its poor soil quality limits crop production and decreases local food supply^17^.

Winter wheat-summer maize double cropping is the main rotation system in this region. Intensive farming not only reduces soil fertility, but also produces superfluous crop straws. Most of the straw produced is burnt in the field or is used as domestic fuel by local farmers^18^. The removal and burning of the straw cause greenhouse gas emissions (CO_2_), atmospheric pollution, and soil nutrient depletion.

Various management practices have been developed to mitigate the negative impact of frequent crop planting. These practices are based on conservation tillage with straw mulching instead of conventional tillage systems^19^. However, in no-tillage systems, the straw decomposition rates (especially maize straw) and nutrient release were slow in the field. Furthermore, long-term no-tillage in this type of soil increases soil bulk density, leads to a shallow plow layer, and accelerates nutrient stratification at the soil surface where residues accumulate over time^17^. Therefore, further optimization of tillage practice and residue management is essential if soil nutrient status and crop production are to be improved in this low-productivity soil.

The objectives of this study were (i) to assess the impacts of different tillage and residue management systems on the amounts and distributions of soil total N and the labile N fractions at six soil depths (0–10 cm, 10–20 cm, 20–30 cm, 30–40 cm, 40–50 cm, and 50–60 cm) under the wheat-maize double cropping system, and (ii) to identify suitable tillage-residue management systems that improve soil properties, N nutrient status, and grain yields in this low-productivity soil.

## Results

### Soil bulk density

**Figure 1:**
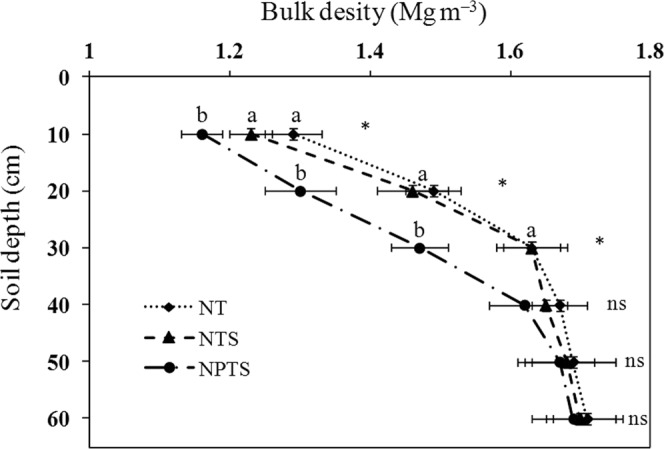
Soil bulk densities at different soil depth under three tillage and crop residue management strategies. Error bars represent standard deviations. Different lower-case letters at the same soil depth indicate significant differences at the 5% level. ns represents no significant differences among the three treatments at the same soil depth. NT: no-tillage with straw removal for winter wheat and summer maize, NTS: no-tillage with straw mulching for winter wheat and summer maize, NPTS: no-tillage with straw mulching for summer maize and plow tillage with straw incorporation for winter wheat.

The soil analysis results indicated that soil bulk density was significantly higher (P < 0.05) in the 0–30 cm soil layer under NT and NTS compared to NPTS (Fig. 1). However, the 0–30 cm layer soil bulk density values for NT and NTS were similar. The soil bulk densities in NPTS were 10.0%, 12.8% and 9.8% less for the 0–10, 10–20, and 20–30 cm layers, respectively, than the values recorded for the NT treatment. Soil bulk density gradually rose as the soil depth increased, regardless of tillage practice and straw management treatments (Fig. 1). There was no apparent difference in soil bulk density among the three treatments in the 30–60 cm layer.

### Nitrogen inputs, TOC (total organic C), and TN (total N)

The actual annual N inputs from the crop residue biomasses are summarized in Table 1. The annual N input from the residue biomass (including stubble and roots) into the soil was 25.4 kg N ha^−1^ y^−1^ for NT. The annual N inputs in the straw retention treatments, which included stubble, straw, and roots, were 96.0 kg N ha^−1^ y^−1^ and 89.8 kg N ha^−1^ y^−1^ for NPTS and NTS, respectively (Table 1).

**Table 1:** Crop biomasses and estimated N inputs under the different tillage and crop residue management systems between 2009 and 2017.

Treatment	Mean annual crop biomass (Mg ha^−1^ y^−1^)				Mean annual nitrogen inputs (kg N ha^−1^ y^−1^)				Total N
	Root biomass	Stubble biomass	Straw biomass	Grain yield	Straw-N	Root-N	Stubble-N	Fertilizer-N	
NT	5.6	1.3	12.3	14.1	—	20.9	4.5	465.0	490.4
NTS	6.9	1.5	14.7	14.9	61.5	22.7	5.6	465.0	554.8
NPTS	7.3	1.7	15.9	16.2	64.3	25.3	6.4	465.0	561.0

NT: no-tillage with straw removal for winter wheat and summer maize, NTS: no-tillage with straw mulching for winter wheat and summer maize, NPTS: no-tillage with straw mulching for summer maize and plow tillage with straw incorporation for winter wheat.

**Figure 2:**
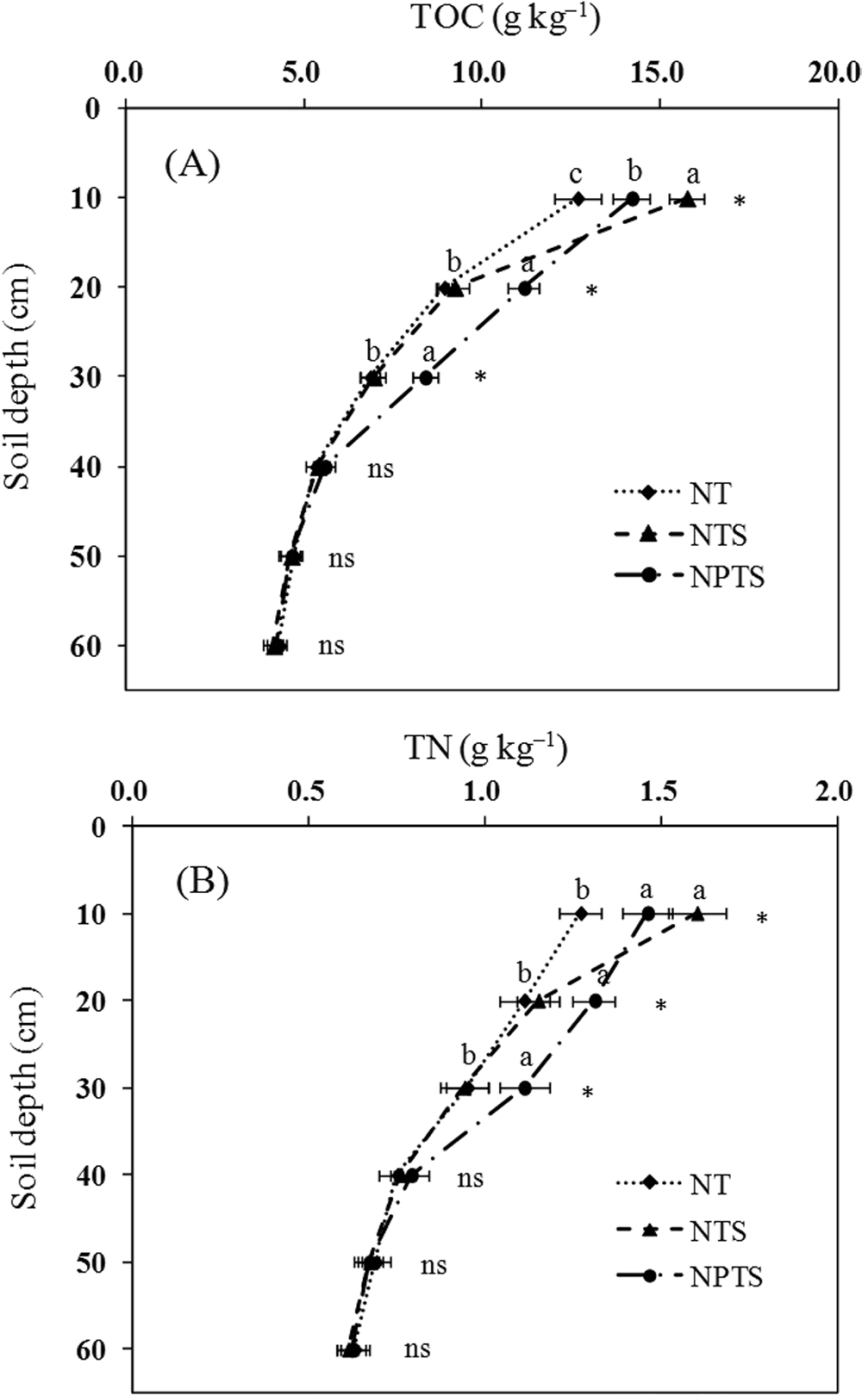
Soil total organic C (TOC) (**A**) and total N (TN) (**B**) at different soil depths for the different tillage and crop residue management strategies. Error bars represent standard deviations. Different lower-case letters for the same soil depth indicate significant differences at the 5% level. ns represents no significant differences among the three treatments at the same soil depth. NT: no-tillage with straw removal for winter wheat and summer maize, NTS: no-tillage with straw mulching for winter wheat and summer maize, NPTS: no-tillage with straw mulching for summer maize and plow tillage with straw incorporation for winter wheat.

The sequence for TOC content in the 0–10 cm layers was NTS > NPTS > NT (Fig. 2A). In the 10–30 cm soil layer, NPTS had a higher TOC content than NT and NTS, which increased by 25.2% and 21.2% in the 10–20 cm layer compared to NT and NTS, respectively. The corresponding percentages were 22.6% and 21.1% in the 20–30 cm layer. The TOC content decreased as depth increased and there were no significant differences in TOC contents between the three treatments at the deeper soil depths (40–60 cm) (Fig. 2A).

The TN contents under the NTS and NPTS treatments at 10 cm depth were significantly (P < 0.05) greater than under the NT treatment (Fig. 2B). However, there were no significant TN content differences between NT and NTS for the 10–30 cm layer. The results also showed that NPTS increased the TN content compared to NT and NTS in the 10–20 cm layer by 18.0% and 13.9%, and in the 20–30 cm layer by 16.8% and 18.1%, respectively (Fig. 2B). There were no significant differences in TN content among the three different treatments for the deeper layers (40–60 cm). These results demonstrated that tillage practice and residue management influenced the TOC and TN contents, but that their effects were mainly restricted to the upper layers (0–30 cm) (Fig. 2).

### Soil N fractions

**Figure 3:**
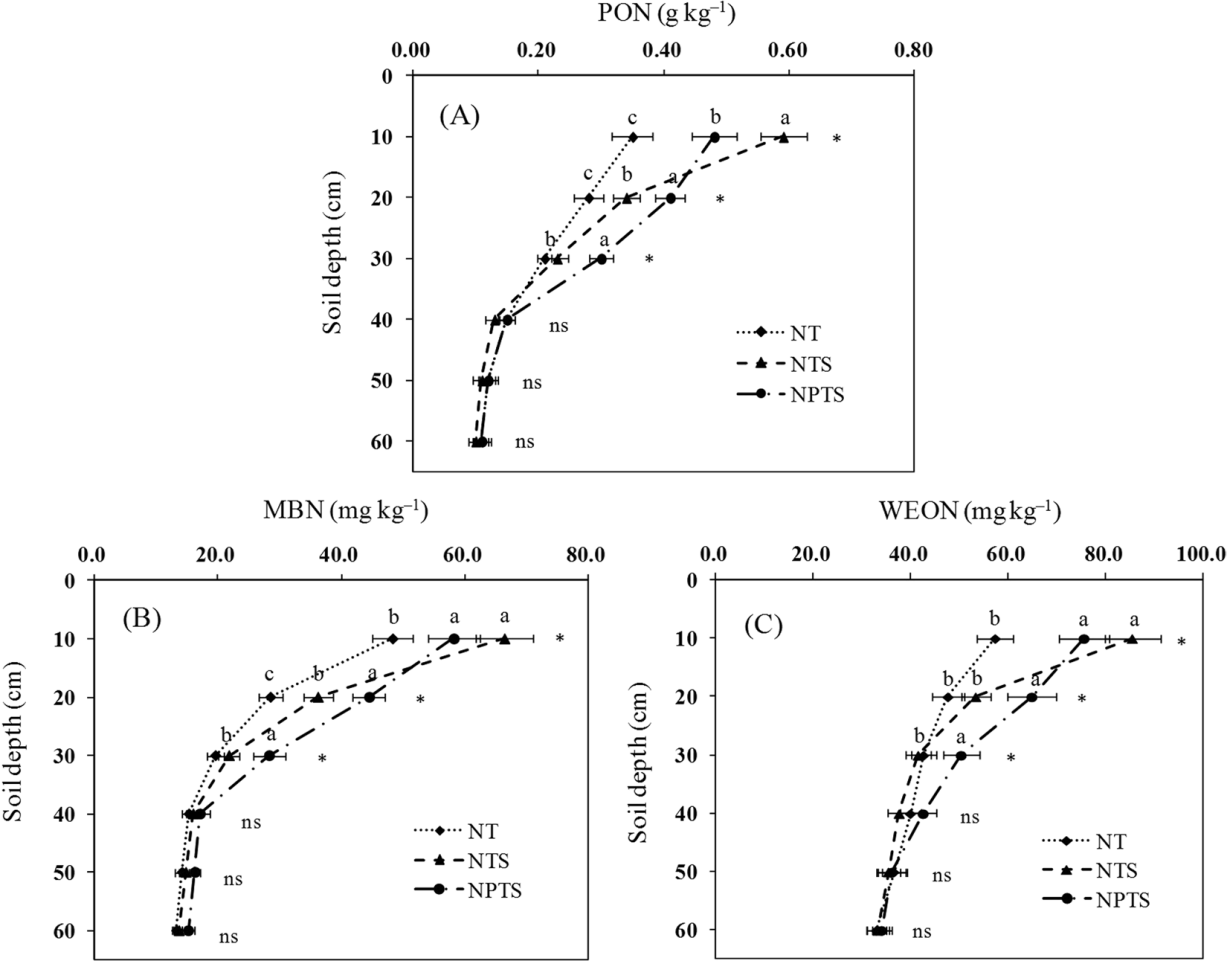
Soil particulate organic N (PON) (**A**), microbial biomass N (MBN) (**B**), and water-extractable organic N (WEON) (**C**) at different soil depths under the different tillage and crop residue management strategies. Error bars represent standard deviations. Different lower-case letters at the same soil depth indicate significant differences at the 5% level. ns represents no significant differences among the three treatments at the same soil depth. NT: no-tillage with straw removal for winter wheat and summer maize, NTS: no-tillage with straw mulching for winter wheat and summer maize, NPTS: no-tillage with straw mulching for summer maize and plow tillage with straw incorporation for winter wheat.

Tillage practice and residue management significantly influenced the PON content (Fig. 3A). The NTS treatment had a particularly significant effect on PON (P < 0.05) in the 0–10 cm soil layer where the increases were 68.6% and 22.9% more than NT and NPTS, respectively. The NPTS treatment had the greatest effect on the PON contents in the 10–30 cm layers, where it increased the PON percentage by 46.4% and 20.6% in 10–20 cm layer, and 42.9% and 30.4% in 20–30 cm layer, respectively, compared to NT and NTS (Fig. 3A). No significant differences were observed below 30 cm.

In the upper soil layer (0–10 cm depth), NTS contained more MBN (66.4 mg kg^−1^) than NPTS (58.2 mg kg^−1^) and NT (48.3 mg kg^−1^) (Fig. 3B). The MBN contents declined as the soil depth increased, but to different extents. The MBN contents in NPTS were significantly higher (P < 0.05) than NT and NTS in the 10–20 cm and 20–30 cm layers, respectively (Fig. 3B). However, there were no significant differences in MBN contents between the NT and NTS treatments for all depths below 20 cm.

Straw retention increased the WEON contents in the 0–10 cm soil layer compared to straw removal (Fig. 3C). The NPTS treatment significantly increased WEON contents compared to NT and NTS by 37.3% and 21.6% in the 10–20 cm layer, and by 18.1% and 21.2% in the 20–30 cm layer, respectively. However, there were no significant differences between the three treatments (P > 0.05) for the deeper layers (40–60 cm).

### Soil mineral N

**Figure 4:**
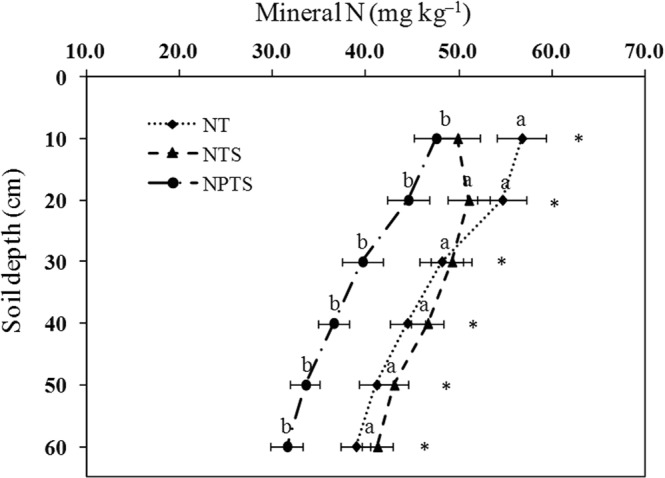
Mineral N contents at different soil depths under the different tillage and crop residue management strategies. Error bars represent standard deviations. Different lower-case letters at the same soil depth indicate significant differences at the 5% level. ns represents no significant differences among the three treatments at the same soil depth. NT: no-tillage with straw removal for winter wheat and summer maize, NTS: no-tillage with straw mulching for winter wheat and summer maize, NPTS: no-tillage with straw mulching for summer maize and plow tillage with straw incorporation for winter wheat.

The NTS treatment had a significantly lower (P < 0.05) mineral N content than NT in the 0–10 cm layer, but there were no significant differences between NTS and NPTS (Fig. 4). The results for the 10–60 cm soil layers showed that mineral N contents in NPTS were markedly lower (P < 0.05) than in NT and NTS (Fig. 4). This indicated that NPTS treatment could immobilize the mineral N in 10–30 cm layer, and reduced leaching losses into deeper soil layers (40–60 cm).

### Correlation between TN and its fractions

Total nitrogen was positively correlated with the soil N fractions (Table 2). The MBN was most highly correlated with TN, followed by PON and WEON. The PON, MBN, WEON, and mineral N were significantly and positively correlated with each other.

**Table 2:** Correlation coefficients between TN and its fractions.

Parameter	TN	PON	MBN	WEON	Mineral N
TN	1				
PON	0.964^**^	1			
MBN	0.989^**^	0.968^**^	1		
WEON	0.956^**^	0.945^**^	0.976^**^	1	
Mineral N	0.607^**^	0.545^*^	0.535^*^	0.478^*^	1

TN: soil total nitrogen, PON: particulate organic nitrogen, MBN: microbial biomass nitrogen, WEON: water-extractable organic nitrogen. ^**^Significant at P < 0.01, ^*^Significant at P < 0.05.

### Stratification ratio for TN and TOC

**Figure 5:**
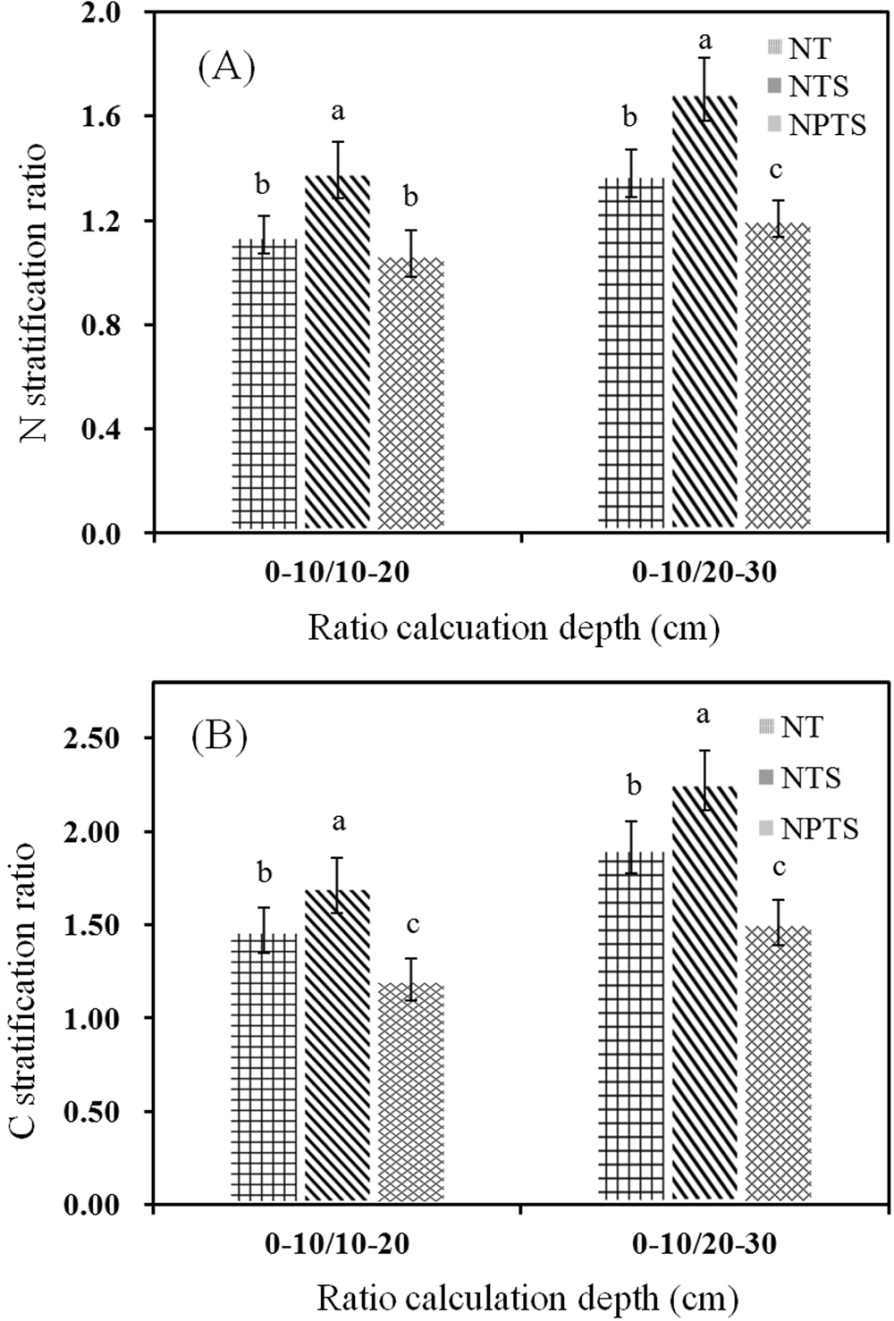
Soil Nitrogen (N) (**A**) and carbon (**C**) (**B**) stratification under the different tillage and crop residue management strategies. Different lower-case letters at the same soil depth indicate significant difference at the 5% level. ns represents no significant differences among the three treatments at the same soil depth. NT: no-tillage with straw removal for winter wheat and summer maize, NTS: no-tillage with straw mulching for winter wheat and summer maize, NPTS: no-tillage with straw mulching for summer maize and plow tillage with straw incorporation for winter wheat.

The stratification ratio (SR) was calculated by dividing the TN or TOC contents in the soil surface layer (0–10 cm) with the corresponding values in lower layers (10–20 and 20–30 cm)^20^. The SR for TN in the 0–30 cm layer was significantly lower (P < 0.05) under NPTS compared to NT and NTS, and the sequence was NPTS < NT < NTS (Fig. 5A). The SR trend for TOC was similar to TN in the 0–30 cm layer (Fig. 5B). The maximum SR was observed under NTS in the 0–10 cm and 20–30 cm layers. These results indicated that NPTS could alleviate TOC and TN stratification in both the surface and subsurface layers (0–30 cm).

### Crop grain yield

**Figure 6:**
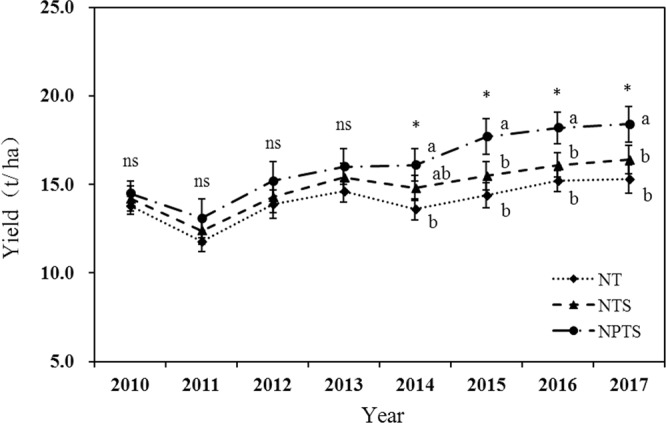
Yield trends for winter wheat and summer maize under the different tillage and crop residue management strategies. Different lower-case letters at the same soil depth indicate significant differences at the 5% level. ns represents no significant differences among the three treatments at the same soil depth. NT: no-tillage with straw removal for winter wheat and summer maize, NTS: no-tillage with straw mulching for winter wheat and summer maize, NPTS: no-tillage with straw mulching for summer maize and plow tillage with straw incorporation for winter wheat.

The total yields for wheat and maize in the three treatments fluctuated widely from year to year (Fig. 6). The NPTS treatment maintained tvhe higher wheat and maize yields than the other treatments in each year from 2015 to 2017 (Fig. 6). The NPTS treatment produced the largest grain yield, and the mean crop yield for NPTS was 14.7% and 8.5% greater than for NT and NTS, respectively, over the 8 years. However, there were no significant differences in crop yield among the other treatments in 2010–2017.

## Discussion

### Soil bulk density

The results showed that bulk density in the 0–30 cm layer was lower under NPTS than under NT and NTS (Fig. 1). This was probably due to loosening of the soil by the tillage operation, which mixed the crop residues into the plow layer^21^. Reductions in soil bulk densities have been reported previously when the soil is tilled or there is a straw retention management program^18^. The decline in bulk density tends to increase soil total porosity^22^, which means that the NPTS treatment could alter the soil structure, leading to decreased soil compaction in the 0–30 cm soil layer.

### TOC and TN

In this study, the TOC and TN content in NTS was greater in the 0–10 cm layer than in NT and NPTS (Fig. 2). However, the TOC and TN content decreased rapidly in NTS as the soil depth increased. The strong stratification of TOC and TN in the top layers of the NTS-treated soil (Fig. 5), which was mainly due to surface residue mulching, was in accordance with many other studies^23^. However, the results from this study showed that the TOC and TN contents in the 10–30 cm layers were higher under NPTS than under NT and NTS. This was mostly due to deep burial of the straw, stubble and root residues in the subsurface soil^24,25^. These results indicated that the distribution and accumulation of TOC and TN was affected by tillage and residue management practices. The lower TOC and TN contents in the deeper soil profile (30–60 cm) under all treatments (Fig. 2) were probably due to there being no crop residue input.

### PON, MBN and WEON

In this study, NTS had a higher PON, MBN and WEON in the 0–10 cm layer, which was probably due to the consecutive wheat and maize straw cover^26^. The PON, MBN and WEON content in the NPTS subsurface soil layer (10–30 cm) was greater than in NTS and NT (Fig. 3B,C). The incorporation of crop residues could act as a cementing agent, which helps stabilize macroaggregates and protect intra-aggregate N in the form of PON^27^. The microorganism activities were increased by carbon source inputs from crop residues^6^. Tillage enhances subsoil aeration, which also increases microbial activity^28^. Some research has demonstrated that the decomposition of crop residues could lead to higher WEON values in the soil^29^. The higher PON, MBN and WEON contents in the NPTS subsurface soil (10–30 cm) compared to NT and NTS were possibly due to the decomposition of maize residues in the subsurface soil and their translocation from the surface soil^30^.

### Soil mineral N

The tillage practices and straw management regimes had significant effects on the distribution of mineral N in the soil profile. These results indicated that the mineral N contents in the 0–10 cm soil layer under NTS and NPTS were significantly less than for the NT treatment (Fig. 4). In addition, the mineral N content was clearly lower in NPTS between 10 and 60 cm depth compared to NT and NTS. There are three possible reasons for the low mineral N content in NPTS-treated soils. Firstly, the maize residue could effectively immobilize the mineral N in soils due to the high C:N ratio and the greater lignin and polyphenol contents^31^. Secondly, straw incorporation promoted the microbial immobilization of applied N and effectively decreased N losses in the soils^32^. This suggestion would support Pisani^33^ who reported that the soil mineral N content was primarily controlled by microbial C and N cycling processes in soils. Thirdly, the soil mineral N content was lower because crop growth in the NPTS treatment requires more soil mineral N, as noted in Dong^34^. Therefore, NPTS effectively regulated N availability and reduced mineral N leaching loss into the deeper soil layers.

### Yield performance

The NPTS treatment increased wheat and maize yields, especially after five years (Fig. 6). The significant crop yield improvement observed in NPTS was attributed to the combined effect of tillage practice and crop residue management on reducing soil bulk density and penetration resistance, which improved the soil properties in the tilled layer. This would lower the stratification of soil nutrients and increase nutrient utilization efficiency. In contrast, the higher bulk density under the long-term NT system can lead to soil compaction and a shallow plow layer in soil, which will ultimately affect soil functional properties and consequently crop growth. Some studies have reported that subsoiling can significantly increase crop grain yields, and nutrient and water use efficiency, compared to no subsoiling under a no-tillage system^35^. A positive yield response to crop straw retention in the wheat–maize cropping system was also found by Dikgwatlhe^36^. N nutrient release from decomposing crop residues could be synchronised with crop demand^37^. The residue decomposition and N nutrient release is thus a pre-requisite for optimising N-use efficiency by crop. The crop yield will depend on the fertilizer value of plant residues left in the soils via their ability to decompose and release N nutrients.

The no-tillage with straw mulching combination adopted in the maize planting season and the plow tillage with straw incorporation used in the wheat planting season could be an effective model. This is possibly because the high temperature and rainfall in summer is beneficial to the decomposition of wheat straw at the soil surface, and burying maize straw in the soil during the cold winter may facilitate the decomposition of maize straw. Therefore, NPTS can be considered to be an important agricultural management practice for improving crop production on a typically low-yielding soil (lime concretion black soil) in central China.

## Conclusions

These results indicated that NPTS significantly reduced soil bulk density in the 0–30 cm soil layer and increased TN, PON, MBN and WEON contents in the 10–30 cm soil layers compared to NT and NTS. The NPTS treatment reduced the excessive mineral N in the soil surface and sub-surface layers, which led to a decrease in leaching losses. The long-term effect of NPTS also led to a higher crop yield compared to NT and NTS. Therefore, NPTS practice offers a significant benefit to the current farming systems in lime concretion black soil in central China, particularly in Anhui province.

## Methods

### Climate and experimental site

The field experiment was carried out in Linquan County (32°56′N, 115°11′E) in Anhui Province, China. The experimental area has a sub-humid continental monsoon climate, with an annual average temperature of 16.2 °C and an annual average precipitation of 830 mm. The soils in this study belong to a typical lime concretion black soil, which is classified as a Calcic Vertisol soil (WRB 2006). The basic soil properties of the surface soil (0–20 cm depth) at the beginning of the experiment were as follows: pH 6.8, soil organic carbon 10.3 g kg^−1^, total N 0.96 g kg^−1^, total P 0.26 g kg^−1^, and total K 15 g kg^−1^. The soil texture in the plow layer was 32% sand, 25% silt, and 43% clay.

### Experimental designs

The experimental period was from 2009 to 2017 and used a winter wheat-summer maize rotation system. It was a randomized block design with three replicate plots per treatment. The summer maize was planted from June to October and the winter wheat was cultivated from October to June (next year). The wheat variety was Yan Nong 19 and the maize variety was TianTai 16.

The experimental treatments at the research site were (1) no-tillage with straw removal for winter wheat and summer maize (NT), (2) no-tillage with straw mulching for winter wheat and summer maize (NTS), and (3) no-tillage with straw mulching for summer maize and plow tillage (to a depth of approximately 30 cm) with straw incorporation for winter wheat (NPTS). Each of the experimental plots was 600 m^2^ (60 m × 10 m). The chemical fertilizer application rates for each year were 225 kg (N) ha^−1^ y^−1^, 90 kg (P_2_O_5_) ha^−1^ y^−1^, and 90 kg (K_2_O) ha^−1^ y^−1^ for wheat, and 240 kg (N) ha^−1^ y^−1^, 90 kg (P_2_O_5_) ha^−1^ y^−1^, and 90 kg (K_2_O) ha^−1^ y^−1^ for maize. A total of 70% of the N fertilizer and all the P and K fertilizer were applied as a basic fertilizer, and 30% N fertilizer was applied at the elongation stage for wheat and at the V12 stage for maize.

After harvest, crop residues were removed from the field in NT, or chopped twice (5–8 cm long) with a residue chopper in NTS and NPTS so that only a small amount of the standing stubble, with a height of 15–20 cm, remained. Deep tillage used a moldboard plough after planting to a depth of 30 cm, and the maize straw residues were evenly distributed on in the soil (0–30 cm depth).

### Soil sampling and analytical methods

A composite soil sample was collected at six depths (0–10, 10–20, 20–30, 30–40, 40–50, and 50–60 cm) from each plot on June, 2017 (the winter wheat harvest). Each mixed soil sample was divided into two parts after carefully removing the fine roots and impurities from the soil. One part of the soil sample was air-dried to measure the basic soil properties, and the other part was stored as a fresh sample for biochemical analysis.

Soil bulk density was measured by the core ring method^38^. Soil samples were collected at each depth, and oven dried at 105 °C for 24 h to obtain the dry weight.

Soil total N was determined using the Kjeldahl method^39^. Particulate organic N (PON) was analyzed using the following steps^19^: 10 g of air-dried soil was added to 30 mL of 5 g L^−1^ sodium hexametaphosphate solution and shaken for 16 h. The soil was then passed through a 53 μm sieve, and the matter remaining on the screen was dried at 50 °C and weighed. The total N content of the PON was measured by the TN method, as described above.

Mineral N (NH_4_-N and NO_3_-N) was extracted from 10 g of moist soil in 50 mL of 2 mol L^−1^ KCl before filtering^40^. The NH_4_-N and NO_3_-N concentrations in the extract were determined using a continuous flow analyzer (AA3, Bran + Luebbe, Germany). Mineral N is the sum of the NH_4_-N and NO_3_-N contents.

The soil microbial biomass N (MBN) was determined by the fumigation extraction method^41^. Briefly, fresh soil samples (equivalent to 25 g oven-dry weight) were fumigated with chloroform at 25 °C for 24 h. Both non-fumigated and fumigated soil samples were extracted using 100 mL 0.5 M K_2_SO_4_ on a rotary shaker at 220 rpm for 30 min before filtering. Nitrogen in the filtrate was analyzed using dual-wavelength ultraviolet spectrophotometry after alkaline persulfate oxidation^42^. A conversion factor of 0.45 was employed to account for incomplete extraction^43^.

The water-extractable organic N (WEON) content was measured according to^19^. Briefly, 10 g of moist soil was extracted with 50 mL water and shaken at 250 rpm for 1 h. Then the sample was centrifuged at 12,000 rpm for 10 min before passing through a 0.45 μm membrane filter. The total water-extractable N (TWEN) in the filtrate was measured using dual-wavelength ultraviolet spectrophotometry after alkaline persulfate oxidation. Water-extractable inorganic N (NH_4_-N and NO_3_-N) was extracted using the same method and determined using a continuous flow analyzer (AA3, Bran + Luebbe, Germany). The WEON was the difference between the TWEN and water-extractable inorganic N contents.

### Crop residue N inputs

The straw and stubble were collected from three 1 m^2^ areas in each plot immediately after the grain harvest. The root biomass was collected from four soil cores (10 cm diameter by 60 cm depth) per plot (two from the rows and the other two from between the rows) after harvest. The straw, stubble, and root samples were then oven-dried at 60 °C for 72 h and weighed. The oven-dried straw, stubble, and root materials were ground and passed through a 0.25 mm sieve to determine the N content. The contributions made by weed biomass and rhizodeposition to TN were ignored in this study.

### Statistical analysis

Analysis of variance (ANOVA) was used to determine the differences among treatments with separation of means by Duncan’s multiple range at P < 0.05. Pearson’s correlation analysis was employed to determine the relationships between soil parameters. The statistical analysis was performed using the SPSS 16.0 statistical package (SPSS Inc., Chicago, IL, US).
